# Resources to assist in the transition to a single IRB model for multisite clinical trials

**DOI:** 10.1016/j.conctc.2019.100423

**Published:** 2019-07-19

**Authors:** Cynthia Hahn, Petra Kaufmann, Soo Bang, Sara Calvert

**Affiliations:** a Integrated Research Strategy, LLC, United States; b National Center for Advancing Translational Sciences, National Institutes of Health, United States; c Celgene, United States; d Clinical Trials Transformation Initiative, United States

## 1 Introduction

The use of a single institutional review board (sIRB) of record will be required for most federally funded non-exempt multisite research studies conducted in the United States with the now-effective National Institutes of Health (NIH) Final Policy on the Use of a Single Institutional Review Board for Multi-Site Research [1] and the upcoming amendments to the federal human research participant protection regulations (known as the Common Rule) [2]. Resources to assist in the implementation of these new rules are available from ongoing efforts of the National Center for Advancing Translational Sciences (NCATS) and the Clinical Trials Transformation Initiative (CTTI).

## 2 Background

The regulatory requirement for IRB oversight of clinical trials was established to protect research participants, and the business model that developed over the past 40 years to support this goal involved the development of thousands of local IRBs and offices across the United States. As the clinical research enterprise evolved and multisite trials became more common, it was unclear whether the goal of protecting research participants was enhanced by having each site's local IRB conduct a full review of the research study. One intuitive objection to full review by multiple local IRBs is the additional time and expense involved [[3], [4], [5]]. In 2006, a National Conference on Alternative IRB Models was held [6] and the U.S. Food and Drug Administration (FDA) issued a guidance for centralized IRB use for multicenter clinical trials, encouraging use especially in situations where it could improve the efficiency of IRB review [7]. Dr. Jerry Menikoff's editorial in the *New England Journal of Medicine* in 2010 [8] suggested that this might be an ethical issue as well as an efficiency issue because multiple local IRBs reviewing the same multisite study leads to a diffusion of responsibility and potentially exposes trial participants to undue risks. Despite this federal support, local IRBs continued to vary in their willingness to defer to sIRB review for multisite trials [9].

**Fig. 1:**
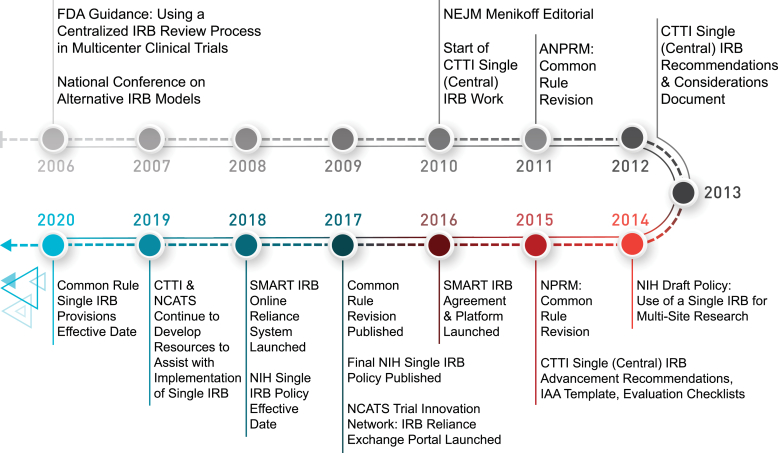
**Timeline of single IRB requirements and release of implementation Resources** Abbreviations: ANPRM = Advance notice of proposed rulemaking; CTTI=Clinical trials transformation initiative; FDA=Food and drug administration; IAA=IRB Authorization agreement; IRB=Instituional review board; NCATS=National center for advancing Translational sciences; NEJM=New england journal of medicine; NIH=National institutes of health; NPRM=Notice of proposed rulemaking; SMART=Streamlined, Multisite, Accelerated resources for trials.

It was in this environment that CTTI launched its first project on the use of a sIRB of record for multisite clinical trials. In 2011, the U.S. Department of Health and Human Services invited commentary on their initial proposed change to the Common Rule to mandate that all domestic sites in a multisite study rely on an sIRB as their IRB of record for that study [10]. Submitted concerns included (1) feasibility of working with multiple external IRBs, each requiring different forms and/or electronic systems to submit a protocol, (2) legal liability and insurance for negligence or events due to errors or omissions by an IRB that is not part of the operations of an organization, and (3) how to pay for and structure an institutional human research protection program that would outsource IRB review but retain institutional oversight [11]. The NIH released a draft policy for comment in 2014, to promote the use of an sIRB of record for domestic sites of multisite studies funded by the NIH [12]. During this period of proposed Common Rule changes and the draft NIH Single IRB policy, NCATS funded a collaborative effort to harmonize and streamline the IRB review process for multisite studies, which became the Streamlined, Multisite, Accelerated Resources for Trials IRB (SMART IRB) Agreement and Platform [13]. CTTI also continued work to address remaining barriers voiced in public comments and in discussions of the first project's results. This paper provides an overview of the efforts and resources created by CTTI and NCATS to assist in the transition to an sIRB model (Fig. 1).

## 3 CTTI single IRB projects and resources

CTTI formed a multistakeholder team in 2010 with representatives from across the clinical trials enterprise to investigate, and propose solutions to, barriers to ceding local IRB review to a designated sIRB for multisite trials. The team followed standard CTTI project methodology [14], which included a literature review, expert advisory panel discussions, interviews with research institutions, and a multistakeholder expert meeting. Full results of the initial project were published in 2013 [9,15] and identified three major concerns: a wide variation in how sIRBs are defined; a conflation of responsibilities between the IRB committee and the institution; and a lack of experience in using an sIRB model. CTTI defined a “central IRB” as a single IRB of record for all sites involved in a multisite protocol.^2^ A range of entities may serve as an sIRB (e.g., another institution's IRB, a federal IRB, an independent IRB). CTTI recommended using an sIRB of record for all sites to improve the quality and efficiency of multisite clinical trials, and for sponsors of multisite networks to require the use of sIRB review in order for relevant stakeholders to gain experience, comfort, and trust with the sIRB review model. To address blurred distinctions between responsibilities for ethics review and other institutional obligations, CTTI developed a Considerations Document [16]. The document supports communication and contractual relationships between institutions and an sIRB by outlining categories of legal and ethical responsibilities to be completed by both the institution and the sIRB, the institution only, the sIRB only, and either the sIRB or the institution.

A common theme encountered after completion of the first project, and in public comments on proposed federal requirements, was that more information and tools were needed to help institutions move forward on adopting an sIRB model [17]. CTTI launched a follow-on project in 2013 to disseminate the first project's results, share examples of sIRB implementation via webinars and presentations at professional conferences, and hold an expert meeting with industry, academia, federal regulators, and other interested parties to discuss remaining barriers [[18], [19], [20]]. Prior to the expert meeting, the multistakeholder team collected example IRB authorization agreements from internet searches and a request on Public Responsibility in Medicine and Research's IRB Forum [21]. This resulted in receipt of 16 template IRB agreements/waivers that were reviewed by the team to determine the kinds of clauses included and the frequency with which those clauses appeared. At the expert meeting, attendees reviewed possible clauses and made decisions about which were essential to include and consensus language for those clauses. The resulting CTTI IRB Authorization Agreement Template provides a standard agreement to address administrative and legal concerns and reduce time when first executing a reliance (authorization) agreement [22]. Expert meeting discussions of common challenges led to the development of the evaluation checklists [23]. The three checklists address institutional readiness to use an sIRB (federal, academic, or independent IRB) for multisite clinical trials; provide general considerations for institutions/sponsors when selecting a particular IRB to serve as the sIRB of record; and provide general considerations for reviewing IRBs when deciding whether to work with a specific institution during a multisite clinical trial.

## 4 NCATS-supported creation of SMART IRB

In 2014, NCATS supported the establishment of a master IRB authorization agreement and standard operating procedures, developed by harmonizing existing sIRB initiatives and by broad input from U.S. medical centers and other stakeholders. The SMART IRB Agreement and Platform were officially launched in 2016, enabling institutions, independent IRBs, and other groups to accept an sIRB reliance agreement template as the platform to cede review to an sIRB without having to negotiate an agreement with each reviewing IRB—thus making it easier for a given institution to rely on several external IRBs [13]. The SMART IRB Online Reliance System launched in 2018, providing a centralized system to request, track, and document reliance arrangements [24]. In addition to the agreement and online reliance system, the SMART IRB website includes templates and harmonized procedures, such as communication plans, fees, and costing models under the NIH policy [25]. The SMART IRB Agreement is also used in the NCATS Trial Innovation Network, which develops systems and best practices to operationalize sIRB review via the IRB Reliance Exchange [26]. The current SMART IRB platform serves to ease common challenges associated with initiating sIRB review of multisite research and to provide a roadmap for institutions to implement the NIH Single IRB Review policy [13].

## 5 Conclusion

The NIH policy requiring an sIRB of record for multisite clinical trials took effect on January 25, 2018; the final changes to the Common Rule will take effect on January 20, 2020 [1,2].

The recommendations and tools generated by CTTI and NCATS provide several resources to assist institutions with the current and upcoming requirements. Because limited published data exist on transitioning from multiple local IRBs to an sIRB, updating the business model for human subject protection programs, and overcoming difficulties, it remains important for IRBs, coordinating centers, and research sites to share best practices with the broader research community [[27], [28], [29]]. Currently, SMART IRB and its Harmonization Steering Committee and the CTTI Single IRB Driving Adoption Committee continue to assess stakeholder needs regarding implementation of sIRB and are creating additional resources to share best practices, improve standardization, and fill remaining information gaps. As all parties gain experience, we believe the sIRB model will ensure participant protections while facilitating more efficient conduct of multisite clinical trials.

## Funding source

Funding for this manuscript and the CTTI Single IRB Projects was made possible, in part, by the United States Food and Drug Administration through grant R18FD005292. The views expressed in written materials or publications and by speakers and moderators do not necessarily reflect the official policies of the Department of Health and Human Services; nor does any mention of trade names, commercial practices, or organization imply endorsement by the United States Government. Partial funding is also provided by CTTI member organizations, as CTTI member organizations pay an annual fee which in a pooled fashion supports CTTI infrastructure expenses and projects.

## Disclaimer

This document represents the opinions of the authors, and it does not represent positions of the National Center for Advancing Translational Sciences (NCATS), the National Institutes of Health (NIH), or of other governmental or regulatory agencies.

## Footnotes

2 CTTI's first two projects used the term “central IRB.” The NIH, as well as current convention, uses the term “single IRB,” abbreviated as sIRB. While CTTI now uses single IRB or sIRB, the terms “central IRB,” “single IRB,” and “reviewing IRB” are synonyms in historical CTTI materials.
